# The Spleen CD4^+^ T Cell Response to Blood-Stage *Plasmodium chabaudi* Malaria Develops in Two Phases Characterized by Different Properties

**DOI:** 10.1371/journal.pone.0022434

**Published:** 2011-07-21

**Authors:** Sandra Marcia Muxel, Ana Paula Freitas do Rosário, Cláudia Augusta Zago, Sheyla Inés Castillo-Méndez, Luiz Roberto Sardinha, Sérgio Marcelo Rodriguez-Málaga, Niels Olsen Saraiva Câmara, José Maria Álvarez, Maria Regina D'Império Lima

**Affiliations:** 1 Departamento de Imunologia, Instituto de Ciências Biomédicas, Universidade de São Paulo, São Paulo, São Paulo, Brazil; 2 Instituto Israelita de Ensino e Pesquisa Albert Einstein, São Paulo, São Paulo, Brazil; Louisiana State University, United States of America

## Abstract

The pivotal role of spleen CD4^+^ T cells in the development of both malaria pathogenesis and protective immunity makes necessary a profound comprehension of the mechanisms involved in their activation and regulation during *Plasmodium* infection. Herein, we examined in detail the behaviour of non-conventional and conventional splenic CD4^+^ T cells during *P. chabaudi* malaria. We took advantage of the fact that a great proportion of CD4^+^ T cells generated in CD1d^-/-^ mice are I-A^b^-restricted (conventional cells), while their counterparts in I-A^b-/-^ mice are restricted by CD1d and other class IB major histocompatibility complex (MHC) molecules (non-conventional cells). We found that conventional CD4^+^ T cells are the main protagonists of the immune response to infection, which develops in two consecutive phases concomitant with acute and chronic parasitaemias. The early phase of the conventional CD4^+^ T cell response is intense and short lasting, rapidly providing large amounts of proinflammatory cytokines and helping follicular and marginal zone B cells to secrete polyclonal immunoglobulin. Both TNF-α and IFN-γ production depend mostly on conventional CD4^+^ T cells. IFN-γ is produced simultaneously by non-conventional and conventional CD4^+^ T cells. The early phase of the response finishes after a week of infection, with the elimination of a large proportion of CD4^+^ T cells, which then gives opportunity to the development of acquired immunity. Unexpectedly, the major contribution of CD1d-restricted CD4^+^ T cells occurs at the beginning of the second phase of the response, but not earlier, helping both IFN-γ and parasite-specific antibody production. We concluded that conventional CD4^+^ T cells have a central role from the onset of *P. chabaudi* malaria, acting in parallel with non-conventional CD4^+^ T cells as a link between innate and acquired immunity. This study contributes to the understanding of malaria immunology and opens a perspective for future studies designed to decipher the molecular mechanisms behind immune responses to *Plasmodium* infection.

## Introduction

Malaria, the infectious disease caused by *Plasmodium* parasites, is a major global health problem that is responsible for the death of over a million people every year [1]. Humans with no previous experience of malaria almost invariably develop a febrile illness that may become severe and lead to death. The asexual blood-stage of the parasite is responsible for the clinical symptoms of the disease. Three overlapping syndromes, severe anaemia, cerebral malaria and respiratory distress, account for most of the severe cases and deaths [2]. Because many of the features of severe malaria are similar to those of sepsis [3], over-vigorous responses to parasites have been implicated in the aetiology of these syndromes [4], [5]. Thus, although TNF-α and IFN-γ appear to be protective against the parasite, very high serum concentrations of proinflammatory cytokines are associated with great morbidity [6], [7]. Yet, exposure to one or two malaria infections appears to be sufficient to induce complete protection from severe illness and death [8], while sterile immunity to malaria parasites is probably never achieved.

Encouragingly, some aspects in mouse models of malaria appear to mirror the human disease with reasonable accuracy. In acutely infected mice, the type 1 responses from CD4^+^ and CD8^+^ T cells are the main participants in the development of both malaria pathogenesis and protective immunity [9], [10], [11]. While TNF-α and IFN-γ are associated with the development of clinical manifestations of the disease [12], [13], [14], the control of acute infection also depends on proinflammatory cytokines [15], [16], [17], which together with acute-phase antibodies [18] may promote parasite clearance by macrophages [19], [20]. CD4^+^ T cells are thought to be essential for complete elimination of the parasite during the late phase of the disease [10], [21], [22], which may also hold true for humans because these cells are critically required to help B cells produce parasite-specific high-affinity immunoglobulin G (IgG) antibodies. However, it is not yet apparent how much of the pathology is dependent on a contribution from the acquired immune response [4].

The pivotal role of CD4^+^ T cells in the development of both malaria pathogenesis and protective immunity makes them putative targets for new strategies to improve the outcome of the disease. On top of being important sources of IFN-γ and helping B cells to secrete antibodies, these cells play a key role in the regulation of immune responses. The acquisition of clinical immunity is likely to be coordinated by CD4^+^ T cells, as clinical immunity remains relatively robust over long periods after being established. The possibility that CD4^+^ T cells are implicated in this process is suggested by the fact that IL-10 or TGF-β deficiency intensifies the clinical signs in mice suffering from malaria [13], [23]. Therefore, a profound comprehension of the mechanisms involved in CD4^+^ T cell activation and regulation during *Plasmodium* infection may improve the chances of developing effective vaccines and other potential immunotherapies to prevent severe malaria syndromes.

CD4^+^ T cells from the spleen, the main lymphoid organ for protection against blood-borne infectious diseases, can be divided into two categories based on how they recognise antigens. Conventional CD4^+^ T cells recognise peptides associated with class II molecules of the major histocompatibility complex (MHC), whereas non-conventional CD4^+^ T cells are a heterogeneous population mostly restricted by class IB MHC molecules [24], which includes CD1d-restricted natural killer T (NKT) cells that recognise lipids [25]. It is generally accepted that non-conventional CD4^+^ T cells are particularly shaped for early immune responses to infections while conventional CD4^+^ T cells are responsible for acquired immunity. Because CD4^+^ T cells from these two categories have been implicated in the immune response to *Plasmodium* parasites [2], we sought to examine in detail the behaviour of non-conventional and conventional splenic CD4^+^ T cells during blood-stage *P. chabaudi* AS strain malaria, a suitable model for the human disease caused by *Plasmodium falciparum* [26]. The information provided here is fundamental for unravelling the molecular mechanisms behind the early phase of the CD4^+^ T cell response to *Plasmodium* infection, knowledge that could help explain why people develop severe malaria and how they get immune against the symptoms of the disease.

## Materials and Methods

### Mice, parasite and infection

Six-to-eight-wk-old C57BL/6 (wild type; WT) (originally from The Jackson Laboratory), CD1d^-/-^ [27] and I-A^b-/-^ (ABBN12) [28] male mice were bred under specific pathogen-free conditions at the Isogenic Mice Facility (Department of Immunology/University of São Paulo, Brazil). CD1d^-/-^ and I-A^b-/-^ mice were used after nine backcrosses onto the C57BL/6 background. *P. chabaudi* AS strain was maintained as described elsewhere [15]. Mice were inoculated intraperitoneally (i.p.) with 1×10^6^ infected red blood cells (iRBC). WT and CD1d^-/-^ mice were challenged i.p. with 1×10^8^ iRBC on day 30 postinfection (p.i.). Parasitaemias were monitored by microscopic examination of Giemsa-stained thin tail blood smears.

### Ethics statement

All procedures were in accordance with national regulations of ethical guidelines for mice experimentation and welfare of the Conselho Nacional de Saúde and Colégio Brasileiro em Experimentação Animal (COBEA) - Brazil, and the protocols were approved by the Health Animal Committee (Comissão de Ética no Uso de Animais – CEUA – ICB/USP) of the Instituto de Ciências Biomédicas of the Universidade de São Paulo, São Paulo, Brazil (permit number 0019/2005 and 0036/2007).

### Spleen cell suspension

Spleen cells were washed and maintained in cold RPMI 1640, supplemented with penicillin (100 U/mL), streptomycin (100 µg/mL), 2-mercaptoethanol (50 µM), L-glutamine (2 mM), sodium pyruvate (1 mM), and 3% heat-inactivated foetal calf serum. All supplements were purchased from Invitrogen Life Technologies. The numbers of cells per spleen were counted using a Neubauer chamber (Sigma-Aldrich).

### Phenotypic analysis of spleen cells

Spleen cells (1×10^6^) were stained with allophycocyanin (APC)-, peridin-clorophyll-protein (PercP)-, fluorescein-isothiocyanate (FITC)-, phycoerythrin (PE)- or CyChrome (Cy)-labelled monoclonal antibodies (mAb) (BD Pharmingen) to CD4 (H129.19), CD8 (53-6.7), CD45R–B220 (RA3-6B2), CD69 (H1.2F3), CD21 (7G6), CD23 (B3B4) and CD1d (1B1) and with the PE-labelled CD1d-α-GalCer tetramer from the NIH (National Institutes of Health - USA) Tetramer Facility. Stained cells were analysed by flow cytometry using a FACSCalibur device with CellQuest software (BD Biosciences).

### IFN-γ intracellular detection

Spleen cells (1×10^6^) were cultured with GolgiStop according to the manufacturer's instructions in the presence of 5 µg/mL plate-bound purified anti-CD3 mAb (145-2C11) or medium alone for 10 h at 37°C in a 5% CO_2_ atmosphere. After washing, cells were surface stained with FITC- or APC-labelled mAb to CD4 and CD8. Cells were then fixed with Cytofix/Cytoperm buffer, stained with PE-labelled mAb to IFN-γ (XMG-1.2) diluted in Perm/Wash buffer, and analysed by flow cytometry. All reagents were purchased from BD Pharmingen.

### CFSE proliferation assay

The proliferative T cell response was measured as previously described [29]. Briefly, spleen cells (3×10^7^) were incubated for 30 min at 37°C with carboxyfluorescein succinimidyl ester (CFSE) (Molecular Probes) (5 µM) in phosphate-buffered saline (PBS) supplemented with 0.1% bovine serum albumin (BSA). Cells (1×10^6^) were then cultured in 96-well plates (Costar) with iRBC (3×10^6^) or medium alone for 72 h at 37°C in a 5% CO_2_ atmosphere. In some experiments, blocking mAb to I-A^b^ (AF6-120.1) and IgG2a isotypic control (G155-178) were added to the cultures (both mAb used at 2.5 µg/mL concentrations). After incubation, cells were stained with PE or APC-labelled mAb to CD4 and CD8 and analysed by flow cytometry.

### Multicytokine assessment

Several cytokines (IFN-γ, TNF-α, IL-2, IL-4 and IL-5) were quantified simultaneously, by the cytometric bead array (CBA; BD Pharmingen), using supernatants obtained from the same cultures used in the CSFE proliferation assay. This technique uses flow cytometry to measure soluble analytes in a particle-based immunoassay and was conducted according to the manufacturer's instructions. The lower limit of detection for all cytokines in this assay was 20 pg/mL.

### Purification of cell subpopulations

Spleen cells (1×10^8^) from CD1d^-/-^ mice were incubated with PE-labelled mAb to CD4 and with anti-PE microbeads diluted in PBS with 0.5% BSA and 2 mM ethylenediaminetetraacetic acid (EDTA) (Invitrogen Life Technologies). Cells were then purified using LS columns (Midi MACS; Miltenyi Biotec). The positive fraction showed > 85% CD4^+^ cells. To obtain spleen cell preparations depleted of dendritic cells (DC), negative selection with anti-Pan DC microbeads (Miltenyi Biotec) was performed, and the negative fraction showed < 0.5% DC (CD11c^+^I-A^b+^ cells).

### Adoptive cell transfers of conventional CD4^+^ cells

WT and I-A^b-/-^ mice received i.p. 4×10^6^ purified CFSE-labelled CD4^+^ cells obtained from CD1d^-/-^ mice. These mice were infected i.p. with 1×10^6^ iRBC on the day of transfer. Proliferative CD4^+^ cell response *in vivo* was analysed on days 4 and 7 of infection. Non-infected mice were used as controls.

### ELISPOT assay for Ig-producing cells

Ig-producing cells were quantified by the ELISPOT assay as previously described [30]. In brief, 96-well flat-bottom microtest plates (Costar) coated overnight (4°C) with goat anti-mouse total Ig (10 µg/mL) were saturated with 1% gelatine (Merck) in PBS for 120 min. Titrated numbers of spleen cells (1×10^6^ to 5×10^2^ cells/well) were cultured for 6 h at 37°C in a 5% CO_2_ atmosphere. The spots were developed by adding goat anti-mouse IgM, IgG2a or IgG1 biotinylated antibodies followed by the addition of a phosphatase alkaline-avidin conjugate (all antibodies and conjugates were purchased from Southern Biotechnology Associates). 5-Bromo-4-chloro-3-indolyl phosphate (BCIP) (Sigma-Aldrich) diluted in 2-amino-2-methyl-1-propanol (AMP) (Merck) was used as the substrate. From the titration plots (number of cells plated *vs.* spots) and the total number of splenocytes, the number of total Ig-producing cells per spleen was calculated.

### ELISA test for parasite-specific antibodies

Anti-*P. chabaudi* antibodies were quantified by ELISA as previously described [31]. In brief, 96-well flat-bottom microtest plates (Costar) were coated overnight (4°C) with a total *P. chabaudi* extract (8 µg/mL). Plates were saturated with 1% BSA for 1 h. After washing, 100 µL of mouse serum samples (diluted from 1/2) was added and left for 90 min at room temperature or overnight (4°C), followed by goat anti-mouse IgG2a and IgG1 peroxidase-conjugated antibodies (Southern Biotechnology Associates) for 1 h. After washing, 100 µL of tetramethylbenzidine (TMB) (Zymed) was added to each well and 15 min later, the absorbance values were quantified by a Spectra Max 190 spectrophotometer (Molecular Devices) with a 650-nm wavelength filter.

### Statistical analysis

Statistical significance was analysed in Graph Pad Prism 4 software using the Mann-Whitney U and Tukey's multiple comparison tests. Differences between groups were considered significant when *p*<0.05 (5%).

## Results

### I-A^b^-restricted CD4^+^ T cells are required for the control of parasitaemia during the early and late *P. chabaudi* infection

**Figure 1:**
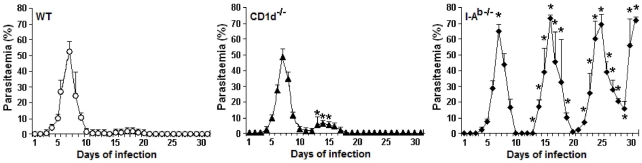
Parasitaemia curves in *P. chabaudi*-infected WT, CD1d^-/-^ and I-A^b-/-^ mice. Mice were infected with 1×10^6^ iRBC. Each curve corresponds to the means ± SD (n = 5–10). *, *p*<0.05, CD1d^-/-^ or I-A^b-/-^ mice compared with WT mice. Data are representative of three experiments.

To evaluate the protective role of CD1d- and I-A^b^-restricted CD4^+^ T cells during early and late *P. chabaudi* malaria, we determined the parasitaemia curves in WT, CD1d^-/-^ and I-A^b-/-^ mice infected with 1×10^6^ iRBC. WT and CD1d^-/-^ mice developed comparable first parasitaemia peaks, but in CD1d^-/-^ mice the second peak was higher and occurred earlier (on days 13 to 15 p.i.) when compared to WT mice (on day 17 p.i.) **(** **Figure 1** **)**. Despite these differences, mice from both groups showed efficient control of parasite growth and survived the infection. Contrarily, I-A^b-/-^ mice developed a significantly higher first peak and prominent recrudescent peaks compared to WT mice. Furthermore, these mice were unable to eliminate the parasite and around 50% were dead one month after infection. These results indicate that parasite control during early and late *P. chabaudi* malaria is dependent on I-A^b^-restricted CD4^+^ T cells, whereas the contribution of CD1d-restricted CD4^+^ T cells is modest and limited to the second parasitaemia peak.

### The majority of spleen CD4^+^ T cells activated during the first days of *P. chabaudi* infection are restricted by class II MHC molecules

**Figure 2:**
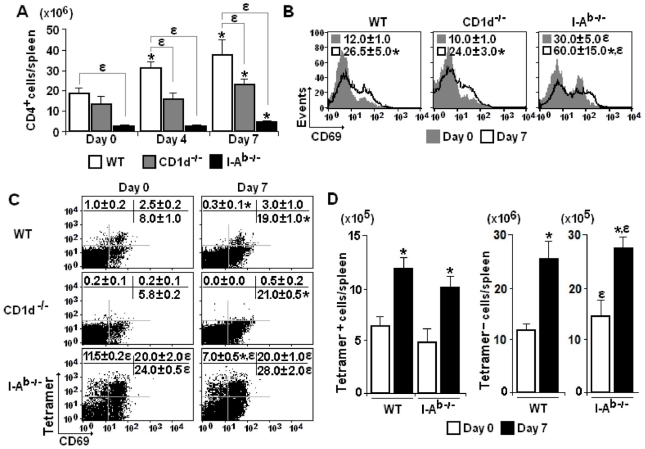
CD4^+^ cell activation in the spleen of *P. chabaudi*-infected WT, CD1d^-/-^ and I-A^b-/-^ mice. (A) Numbers of CD4^+^ cells per spleen on days 0, 4 and 7 of infection. Data represent the means ± SD (n = 6–9). (B) Histograms showing CD69 expression in gated CD4^+^ cells on days 0 and 7 of infection. Numbers in histograms represent the means ± SD (n = 4–6) of MFI values. (C) Dot plots showing gated CD4^+^ cells that recognise CD1d-α-GalCer tetramers and express CD69 molecules on days 0 and 7 of infection. Numbers in dot plots represent the means ± SD (n = 4–6) of the percentages of each subpopulation. (D) Numbers of Tetramer^+^CD4^+^ cells and Tetramer^-^CD4^+^ cells per spleen on days 0 and 7 of infection. Data represent the means ± SD (n = 6). In A–D, *, *p<*0.05, infected mice compared with non-infected mice; *ε*, *p<*0.05, CD1d^-/-^ or I-A^b-/-^ mice compared with WT mice. Data are representative of three experiments.

As the previous results suggest a protective role for I-A^b^-restricted CD4^+^ T cells in early *P. chabaudi* malaria, we set out to evaluate the kinetics of the CD4^+^ T cell response to infection in mice lacking CD1d and I-A^b^ molecules using as parameters spleen cellularity and expression of the early activation marker CD69 [32]. The aim of this analysis was to compare non-conventional and conventional CD4^+^ T cells, which are thought to be primarily involved in innate and acquired immunity, respectively. We took advantage of the fact that a great proportion of CD4^+^ T cells generated in CD1d^-/-^ mice are restricted by I-A^b^ molecules (conventional CD4^+^ T cells), whereas their counterparts in I-A^b-/-^ mice are mostly restricted by CD1d molecules (non-conventional CD4^+^ T cells). According to our data, the lack of CD1d molecules led to reduced numbers of CD4^+^ cells per spleen in infected mice **(** **Figure 2A** **)**. Although the CD4^+^ cell population was notably smaller in I-A^b-/-^ mice compared to WT mice, it also expanded from day 0 to 7 p.i. when maximum cellularity was attained in WT mice. Regarding the CD69 expression, the mean fluorescence intensity (MFI) was considerably higher in CD4^+^ cells in all groups of infected mice compared to controls **(** **Figure 2B** **)**. However, for CD4^+^ cells generated in the absence of I-A^b^ molecules, a two-fold increase in CD69 expression was already observed in non-infected mice (compared to non-infected WT mice), which is a known feature of NKT cells [25], and infection led to additional augmentations of these values. It is worth noting that the absence of αβ T cells did not result in compensatory increase of γδ T cells since less than 1% of CD4^+^ cells in I-A^b-/-^ mice expressed TCRγδ, a proportion similar to that of WT mice, and these percentages were not modified by the infection **(data not shown)**.

To further characterise CD4^+^ T cells responding to *P. chabaudi* infection in CD1d^-/-^, I-A^b-/-^ and WT mice, these cells were analysed in relation to their ability to recognise the CD1d-α-GalCer tetramer. As expected, CD1d^-/-^ mice were deficient in CD4^+^ cells that recognised the tetramer **(** **Figure 2C** **)**. In addition, the majority of CD4^+^ cells expressing CD69 in 7-day infected WT mice were not specific for CD1d-α-GalCer molecules. Confirming the notion that a large proportion of CD4^+^ cells generated in I-A^b-/-^ mice were restricted by CD1d molecules, 31.5% of CD4^+^ cells recognised CD1d-α-GalCer molecules, compared to 3.5% in WT mice. However, the percentages of CD1d-α-GalCer-restricted CD4^+^ cells expressing CD69 in both WT and I-A^b-/-^ mice were not affected by infection. When total numbers of CD4^+^ cells per spleen were considered, on day 7 p.i., there was a 2-fold increase in the Tetramer^+^ and Tetramer^-^ cell populations of WT and I-A^b-/-^ mice **(** **Figure 2D** **)**. Tetramer^-^CD4^+^ cells in I-A^b-/-^ mice may comprise CD1d-restricted CD4^+^ T cells with specificities other than α-GalCer and also, CD4^+^ T cells restricted by class IB MHC molecules other than CD1d [24]. It should be noted, however, that the absolute increase was more than 10-fold greater for the Tetramer^-^ cell population in WT mice (14.8×10^6^ cells) compared to the other cell populations (which ranged from 4.7–13.2×10^5^ cells). The fact that Tetramer^+^CD4^+^ cell numbers per spleen are comparable in I-A^b-/-^ and WT mice, showing a similar increase following infection, suggests that the antigen presenting cell (APC) function is preserved in the absence of conventional CD4^+^ T cells. We concluded that both non-conventional and conventional CD4^+^ T cells promptly respond to *P. chabaudi* malaria, but the majority of splenic CD4^+^ T cells activated during the first days of infection are restricted by class II MHC molecules.

### I-A^b^-restricted CD4^+^ T cells proliferate vigorously during the first days of *P. chabaudi* infection

**Figure 3:**
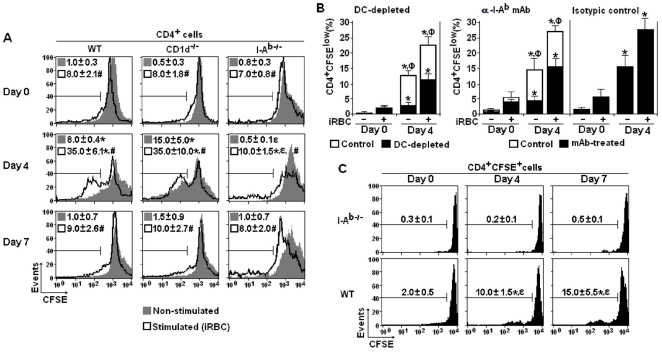
Proliferative response of spleen CD4^+^ cells from *P. chabaudi*-infected WT, CD1d^-/-^ and I-A^b-/-^ mice. (A) Histograms showing spontaneous and iRBC-stimulated *in vitro* proliferation of gated CD4^+^ cells on days 0, 4 and 7 of infection. Numbers in histograms represent the means ± SD (n = 6–8) of the percentages of replicating (CFSE^low^) cells. (B) Effects of DC depletion and anti-I-A^b^ mAb treatment on the *in vitro* proliferation of gated CD4^+^ cells from WT mice on days 0 and 4 of infection. Data represent the means ± SD (n = 6) of the percentages of replicating (CFSE^low^) cells. (C) CFSE-labelled CD4^+^ cells (4×10^6^) from the spleens of CD1d^-/-^ mice were adoptively transferred into WT and I-A^b-/-^ mice. The recipient mice were then infected and the *in vivo* proliferative response was determined 0, 4 and 7 days later. Numbers in histograms represent the means ± SD (n = 6) of the percentages of replicating (CFSE^low^) cells. In A-C, *, *p*<0.05, infected mice compared with non-infected mice. In A and C, *ε*, *p<*0.05, CD1d^-/-^ or I-A^b-/-^ mice compared with WT mice. In A, #, *p*<0.05, spontaneous proliferation compared with iRBC-stimulated proliferation. In B, Ф, *p<*0.05, DC-depleted or mAb-treated cell cultures compared with control cell cultures. Data are representative of three experiments.

As the previous data showed that non-conventional and conventional CD4^+^ T cell populations in the spleen increased with similar kinetics during acute *P. chabaudi* malaria, experiments were then performed to compare the *in vitro* proliferative responses of these cells. Both spontaneous (non-stimulated *in vitro*) and iRBC-stimulated proliferations were evaluated to address the actual and potential proliferative responses, respectively. For CD4^+^ cells obtained from WT and CD1d^-/-^ mice on day 4 p.i., there was a consistent spontaneous proliferation of 8.0% and 15.0% of the cells after 72 h of culture, notably higher than those of non-infected mice (0.5% and 1.0%, respectively) **(** **Figure 3A** **)**. *In vitro* stimulation with iRBC resulted in further increases of these values up to 35.0%. Contrasting with CD4^+^ cells from CD1d^-/-^ mice that proliferated as much as CD4^+^ cells from WT mice, CD4^+^ cells from I-A^b-/-^ mice showed low spontaneous proliferation in 4-day infected mice, with the average frequency of dividing cells increasing from 0.5% to 10.0% following *in vitro* iRBC-stimulation. Interestingly, on day 7 p.i., the spontaneous and iRBC-stimulated proliferation of CD4^+^ cells from WT and CD1d^-/-^ mice was indistinguishable from that in non-infected controls.

To investigate whether the intense proliferative response of spleen CD4^+^ cells from 4-day infected WT and CD1d^-/-^ mice requires interaction with DC and I-A^b^ molecules, DC-depleted cultures and anti-I-A^b^ mAb-treated cultures were compared with positive controls. As shown in **Figure 3B**, in both cases there was considerable reduction in the percentages of proliferating cells. The fact that a significant proliferation is still observed following blockade of I-A^b^- and DC-mediated interactions may result from *in vivo* priming in the presence of I-A^b^ molecules, allowing the TCR signalling and, in consequence, a few rounds of cell proliferation driven by cytokines and costimulatory molecules. To extend this analysis *in vivo*, CFSE-labelled CD4^+^ cells from the spleens of CD1d^-/-^ mice (mostly I-A^b^-restricted CD4^+^ T cells) were adoptively transferred into WT and I-A^b-/-^ mice that were subsequently infected and their proliferative responses were determined 4 and 7 days later. When transferred into infected WT mice, CFSE-labelled CD4^+^ cells rapidly proliferated with 10% and 15% of dividing cells on days 4 and 7 p.i., respectively **(** **Figure 3C** **)**. Corroborating the notion that this response requires interaction with class II MHC molecules, these CD4^+^ cells failed to respond to *P. chabaudi* malaria when transferred into I-A^b-/-^ mice.

### I-A^b^-restricted CD4^+^ T cells are the major source of IFN-γ production in the spleen during the first days of *P. chabaudi* infection

**Figure 4:**
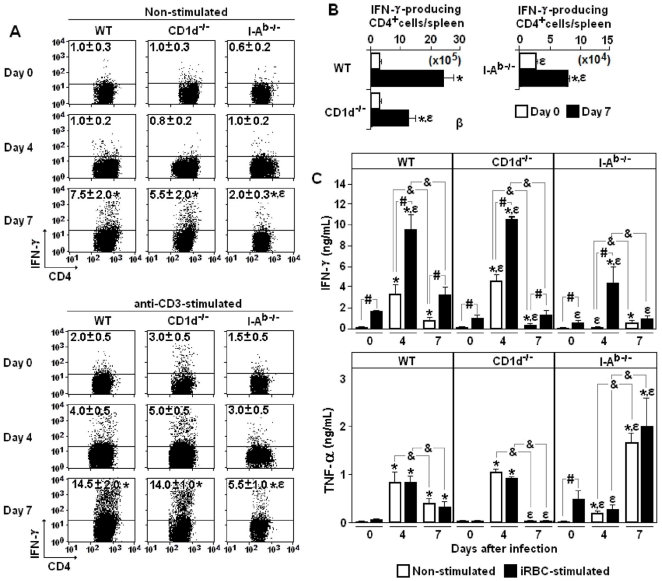
Production of IFN-γ and TNF-α by spleen cells from *P. chabaudi*-infected WT, CD1d^-/-^ and I-A^b-/-^ mice. (A) Dot plots showing intracellular IFN-γ in gated CD4^+^ cells on days 0, 4 and 7 of infection. Non-stimulated and anti-CD3-stimulated cell cultures are shown. Numbers in dot plots represent the means ± SD (n = 4–6) of IFN-γ^+^ cell percentages. (B) Numbers of CD4^+^IFN-γ^+^ cells per spleen on days 0 and 7 of infection. Data represents the means ± SD (n = 4–6). (C) Spontaneous and iRBC-stimulated IFN-γ and TNF-α production in 72-h supernatants from spleens cells on days 0, 4 and 7 of infection. Data represents the means ± SD (n = 6–8). In A-C, *, p<0.05, infected mice compared with non-infected mice; *ε, p*<0.05, CD1d^-/-^ or I-A^b-/-^ mice compared with WT mice. In C, #, *p*<0.05, spontaneous production compared with iRBC-stimulated production; &, *p<*0.05, 4-day infected mice compared with 7-day infected cells. Data are representative of three experiments.

To evaluate the contribution of non-conventional and conventional CD4^+^ T cells to the proinflammatory cytokine response to *P. chabaudi* malaria, IFN-γ production by spleen cells from WT, CD1d^-/-^ and I-A^b-/-^ mice was quantified on days 0, 4 and 7 of infection. *Ex vivo* production of IFN-γ (intracellular staining) was determined in both non-stimulated and anti-CD3 mAb-stimulated cell cultures, which revealed effector cells induced during the acute infection and the total potential of CD4^+^ cells to secrete IFN-γ, respectively. In both cases, the maximum response was observed on day 7 p.i., when the percentages of IFN-γ-producing CD4^+^ cells were 3-fold higher in WT and CD1d^-/-^ mice compared to I-A^b-/-^ mice **(** **Figure 4A** **)**. Interestingly, on day 4 p.i., no significant increase in the percentages of IFN-γ-producing CD4^+^ cells was observed in I-A^b-/-^ mice when compared to non-infected mice. Considering the total cell numbers per spleen on day 7 p.i., WT and CD1d^-/-^ mice showed, respectively, 30- and 15-fold more CD4^+^ cells spontaneously producing IFN-γ compared to I-A^b-/-^ mice **(** **Figure 4B** **)**.

IFN-γ and TNF-α were also quantified *in vitro* in spleen cell supernatants obtained after 72 h of culture (72-h supernatants). For WT and CD1d^-/-^ mice, the spontaneous and iRBC-stimulated production of IFN-γ and TNF-α reached the highest levels in the culture of 4-day infected cells but the values decreased thereafter **(** **Figure 4C** **)**. In contrast, the peak of TNF-α production in cell cultures from I-A^b-/-^ mice occurred with spleen cells from day 7 of infection. Moreover, in I-A^b-/-^ mice, IFN-γ was only detected following iRBC-stimulation of cells from 4-day infected mice, and the levels were 2-2.5-fold lower compared to those of corresponding cells from WT and CD1d^-/-^ mice. The IFN-γ detected in supernatants of 4-day infected I-A^b-/-^ mice is likely to be mainly produced by CD8^+^ cells, which were also an important source of this cytokine as revealed by intracellular staining **(Figure S1)**. Moreover, the fact that CD8^+^ T cells in I-A^b-/-^, CD1d^-/-^ and WT mice show a vigorous IFN-γ response to acute infection corroborates the assumption that the APC function is preserved in these mice. IL-2, IL-4, and IL-5 were also quantified in the same supernatants but the values were below the limit of trustworthy detection **(data not shown)**. In summary, in early *P. chabaudi* malaria, IFN-γ and TNF-α production by spleen cells is intense, short-lasting and mostly dependent on conventional CD4^+^ T cells. These cells are the major source of IFN-γ and show similar kinetics for its production as non-conventional CD4^+^ T cells.

### I-A^b^-restricted CD4^+^ T cells are required for activation of both follicular (FO) and marginal zone (MZ) B cells during early *P. chabaudi* infection

**Figure 5:**
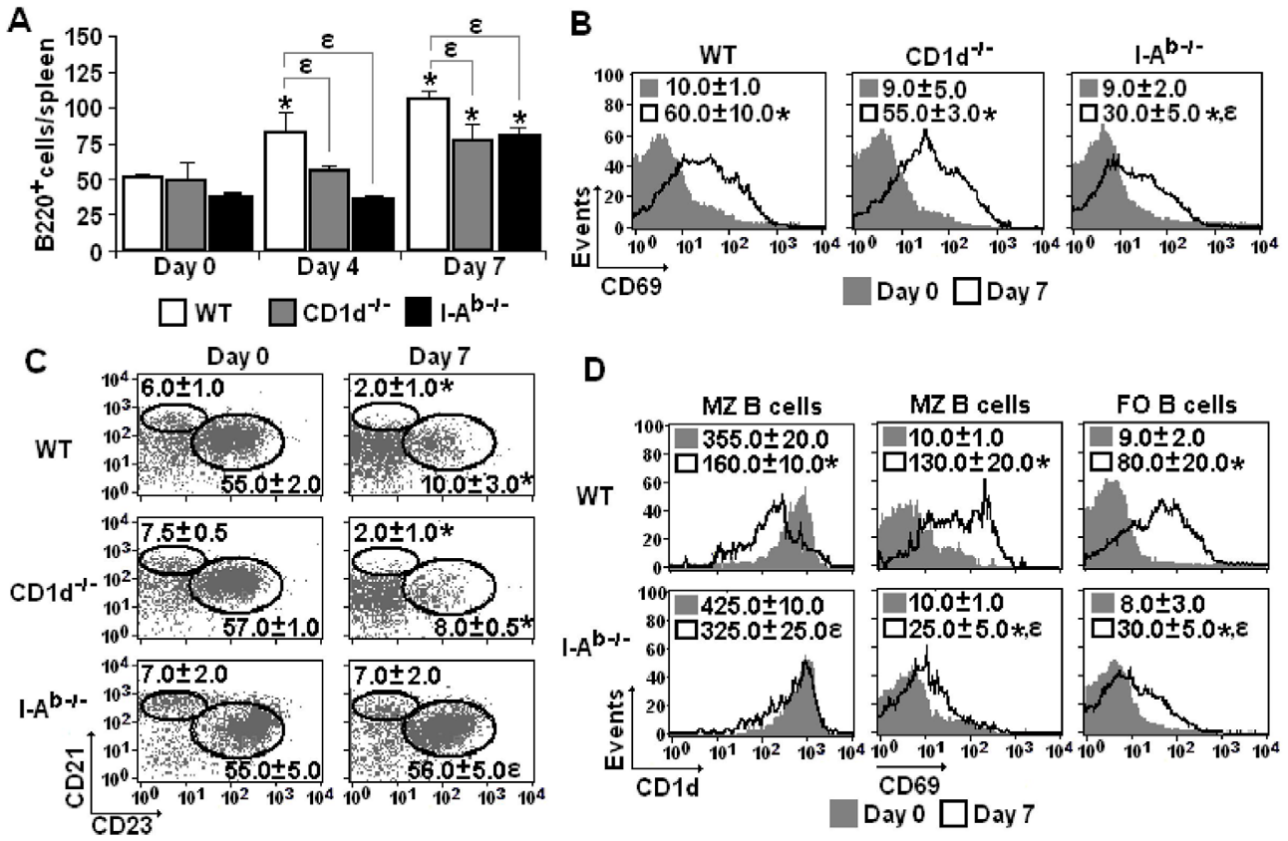
B cell activation in the spleen of *P. chabaudi*-infected WT, CD1d^-/-^ and I-A^b-/-^ mice. (A) Numbers of B220^+^ cells per spleen on days 0, 4 and 7 of infection. Data represent the means ± SD (n = 6–9). (B) Histograms showing CD69 expression in gated B220^+^ cells on days 0 and 7 of infection. Numbers in histograms represent the means ± SD (n = 4–6) of MFI values. (C) Dot plots showing MZ (CD21^high^CD23^low^B220^+^) B cells and FO (CD21^low^CD23^high^B220^+^) B cells on days 0 and 7 of infection. Numbers in dot plots represent the means ± SD (n = 4–7) of the percentages of each subpopulation. (D) Histograms showing CD1d and CD69 expression in gated MZ and FO B cells on days 0 and 7 of infection. Numbers in histograms represent the means ± SD (n = 4–7) of MFI values. In A-D, *, *p*<0.05, infected mice compared with non-infected mice; *ε*, *p*<0.05, CD1d^-/-^ or I-A^b-/-^ mice compared with WT mice. Data are representative of three experiments.

The involvement of non-conventional and conventional CD4^+^ T cells in the early polyclonal B cell response to *P. chabaudi* malaria was then investigated by analysing spleen B cells according to cellularity and CD69 expression on days 0, 4 and 7 of infection. As we have previously shown that, on days 0 and 7 p.i., 90–95% of splenic cells expressing high levels of B220 (CD45R) were sIg^+^ B cells [33], we decided to analyse the behaviour of this population during infection. On day 7 p.i., CD1d^-/-^ and I-A^b-/-^ mice showed reduced, but statistically significant, augmentation of B cell numbers per spleen, when compared to WT mice **(** **Figure 5A** **)**. Data showing the expansion of the B cell population in 7-day infected I-A^b-/-^ mice indicate that at least a proportion of these cells proliferate without help from conventional CD4^+^ T cells. This idea was corroborated by analysis of CD69 expression in B cells from 7-day infected I-A^b-/-^ mice, which showed a lower but statistically significant increase in MFI values compared to B cells from 7-day infected WT and CD1d^-/-^ mice **(** **Figure 5B** **)**. Theoretically, this B cell response in the absence of help from I-A^b^-restricted CD4^+^ T cells could either be T-cell independent or dependent of CD1d-restricted CD4^+^ T cells. The latter possibility could be particularly relevant for MZ B cells that express high levels of CD1d on the surface [34] and are activated during acute *P. chabaudi* malaria [33], [35].

To verify to what extent non-conventional and conventional CD4^+^ T cells are required for the initial steps of FO and MZ B cell responses to *P. chabaudi* malaria, spleen B cells from WT, CD1d^-/-^ and I-A^b-/-^ mice were evaluated on days 0 and 7 p.i. according to CD1d, CD21, CD23 and CD69 expression. While infection led to downregulation of CD21 expression in MZ (CD21^high^CD23^low^B220^+^) B cells and of CD23 expression in FO (CD21^low^CD23^high^B220^+^) B cells from WT and CD1d^-/-^ mice, these phenomena were not observed in B cells from I-A^b-/-^ mice **(** **Figure 5C** **)**. CD1d expression was also reduced in MZ B cells from 7-day infected WT mice but not in those from 7-day infected I-A^b-/-^ mice **(** **Figure 5D** **)**. In addition, on day 7 p.i., the increase of CD69 expression in both MZ and FO B cells was considerably higher in WT mice than in I-A^b-/-^ mice. Based on the B cell numbers per spleen, we concluded that CD1d-restricted and I-A^b^-restricted CD4^+^ T cells contribute to the expansion of the spleen B cell population during early *P. chabaudi* malaria. However, I-A^b^-restricted CD4^+^ T cells are essential for the full activation and phenotypic modifications observed in both MZ and FO B cells.

### Production of polyclonal and parasite-specific Ig during early and late *P. chabaudi* infection is mostly dependent on I-A^b^-restricted CD4^+^ T cells

**Figure 6:**
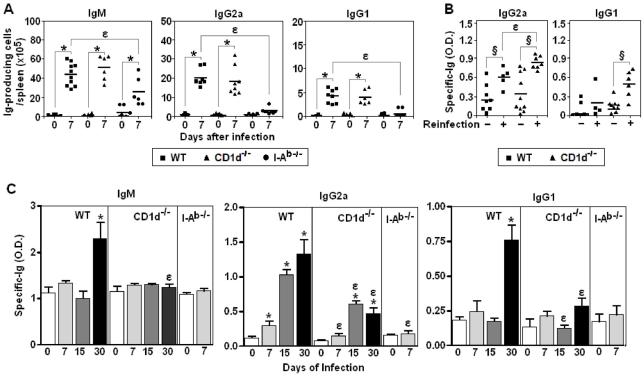
Polyclonal and parasite-specific antibody responses in *P. chabaudi*-infected WT, CD1d^-/-^ and I-A^b-/-^ mice. (A) Total numbers of IgM-, IgG1- and IgG2a-producing cells in the spleen on days 0 and 7 of infection. Each point corresponds to a single mouse. Horizontal lines represent the means of numbers of Ig-producing cells per spleen (n = 6–8). (B) On day 30 p.i., mice were reinfected with 1 × 10^8^ iRBC. Non-reinfected mice were used as controls. The serum levels of parasite-specific IgG2a and IgG1 antibodies were measured 15 days after re-infection (on day 45 p.i.). To favour high-affinity Ig binding, serum samples were incubated with plate-bound parasite antigens during a short period of time (90 min). Each point corresponds to a single mouse. Horizontal lines represent the means of O.D. values (n = 5–10). (C) Serum levels of parasite-specific IgM, IgG2a and IgG1 antibodies were measured on days 0, 7, 15 and 30 of infection. To favour low-affinity Ig binding, serum samples were incubated with plate-bound parasite antigens during a long period of time (overnight). Data represents the means ± SD (n = 4–8). In A, *, *p*<0.05, infected mice compared with non-infected mice. In A and B, *ε*, *p*<0.05, CD1d^-/-^ or I-A^b-/-^ mice compared with WT mice. In B, **§**, *p*<0.05, reinfected mice compared with non-reinfected mice. Data in panel B were statistically different from those of non-infected mice, with the exception of IgG1 O.D. values in non-reinfected mice. Data are representative of three experiments.

Next, experiments were performed to evaluate the involvement of non-conventional and conventional CD4^+^ T cells in polyclonal and parasite-specific Ig responses to *P. chabaudi* infection. We observed that on day 7 p.i., the numbers of total IgM-, IgG2a- and IgG1-producing cells per spleen increased similarly in WT and CD1d^-/-^ mice, whereas in I-A^b-/-^ mice there was only a small augmentation of IgM-producing cells **(** **Figure 6A** **)**. When WT and CD1d^-/-^ mice were reinfected with 1×10^8^ iRBC on day 30 p.i., the parasite-specific IgG2a and IgG1 responses measured 15 days after reinfection (on day 45 p.i.) were higher in mice lacking CD1d-restricted CD4^+^ T cells **(** **Figure 6B** **)**. Since O.D. values were still very low on day 30 p.i., making it difficult to compare the antibody response at earlier times p.i., serum samples were incubated overnight instead of 90 min to improve detection of low-affinity Ig. With this approach, WT mice showed significantly higher levels of parasite-specific IgM (day 30 p.i.), IgG2a (days 7, 15 and 30 p.i.) and IgG1 (day 30 p.i.) compared to CD1d^-/-^ mice **(** **Figure 6C** **)**. Taken together, these results indicate that I-A^b^-restricted CD4^+^ T cells have a central role in the polyclonal Ig response induced by the infection, while CD1d-restricted CD4^+^ T cells contribute to parasite-specific low-affinity antibody response but are not required for production of high-affinity antibodies.

### The early CD4^+^ T cell response to *P. chabaudi* infection is followed by the development of acquired immunity

**Figure 7:**
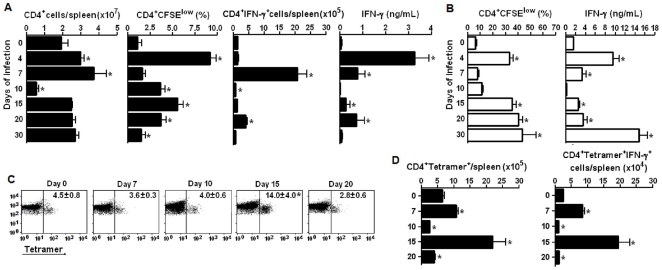
Characterization of CD4^+^ and Tetramer^+^CD4^+^ cell populations in the spleen of WT mice during early and late *P. chabaudi* malaria. (A) Numbers of CD4+ cells per spleen, spontaneous CD4+ cell proliferation and IFN-γ production on days 0, 4, 7, 10, 15, 20 and 30 of infection. Data represent the means ± SD (n = 6–10). CD4+ cell proliferation is represented as percentages of replicating (CFSE^low^) cells. Intracellular and secreted IFN-γ was quantified in gated CD4^+^ cells and in 72-h supernatants, respectively. (B) iRBC-stimulated CD4+ cell proliferation and IFN-γ production in the same groups of mice (n = 6–10). CD4+ cell proliferation is represented as percentages of replicating (CFSE^low^) cells. Secreted IFN-γ was quantified in 72-h supernatants. (C) Dot plots showing gated CD4^+^ cells that recognise CD1d-α-GalCer tetramers (Tetramer^+^ cells). Numbers in dot plots represent the means ± SD (n = 6) of the percentages of Tetramer^+^ cells in gated CD4^+^ cells. (D) Numbers per spleen of Tetramer^+^CD4^+^ cells and Tetramer^+^CD4^+^IFN-γ cells on days 0, 7, 10, 15 and 20 of infection. Data represent the means ± SD (n = 6). In A, B and C, *, *p*<0.05, infected mice compared with non-infected mice. Data are representative of three experiments.

The picture emerging from this study is that soon after parasite inoculation, a substantial proportion of conventional CD4^+^ T cells from the spleen proliferate vigorously and then produce large amounts of IFN-γ and help MZ and FO B cells to produce polyclonal Ig. To evaluate the spleen CD4^+^ T cell population throughout the different phases of *P. chabaudi* malaria, the cell numbers per spleen, spontaneous and iRBC-stimulated proliferation and IFN-γ production were analysed during the first month of infection. As shown in **Figure 7A**, on day 10 p.i., the numbers of CD4^+^ cells per spleen were abruptly reduced and reached values lower than half of those found in non-infected mice. The normalisation of this population occurred concomitantly with a second wave of spontaneous CD4^+^ cell proliferation that peaked on day 15 p.i. and decreased thereafter. The spontaneous IFN-γ production by CD4^+^ cells and total cells also declined on day 10 p.i., although a small augmentation was observed on day 20 p.i. concomitant with the second parasitaemia peak. When stimulated *in vitro* with iRBC, CD4^+^ cells showed an impaired ability to proliferate (from days 7-10 p.i.) and to produce IFN-γ (from days 7–20 p.i.) **(** **Figure 7B** **)**. However, after these periods of CD4^+^ cell unresponsiveness, a huge CD4^+^ cell response was observed, reaching its maximum on day 30 of infection. Unexpectedly, Tetramer^+^ cell percentages within the CD4^+^ cell population increased considerably on day 15 p.i., but not earlier **(** **Figure 7C** **)**. Therefore, while the maximum response of conventional CD4^+^ cells was achieved on day 7 p.i., the peak of the Tetramer^+^ cell numbers per spleen and IFN-γ production occurred on day 15 p.i. **(** **Figure 7D** **)**. At that time p.i., the Tetramer^+^ cell population was a major source of IFN-γ production. These data suggest that the CD4^+^ T cell response to *P. chabaudi* malaria develops in two different phases. The first phase occurs during acute parasitaemia, rapidly generates high numbers of effector cells and abruptly ends when most CD4^+^ cells are eliminated. The second phase begins after acute parasitaemia is controlled and originates a large pool of CD4^+^ T cells with the ability to promptly proliferate and produce IFN-γ in response to iRBC. The major contribution of IFN-γ-producing iNKT cells is observed at the beginning of the second phase of the response.

## Discussion

**Figure 8:**
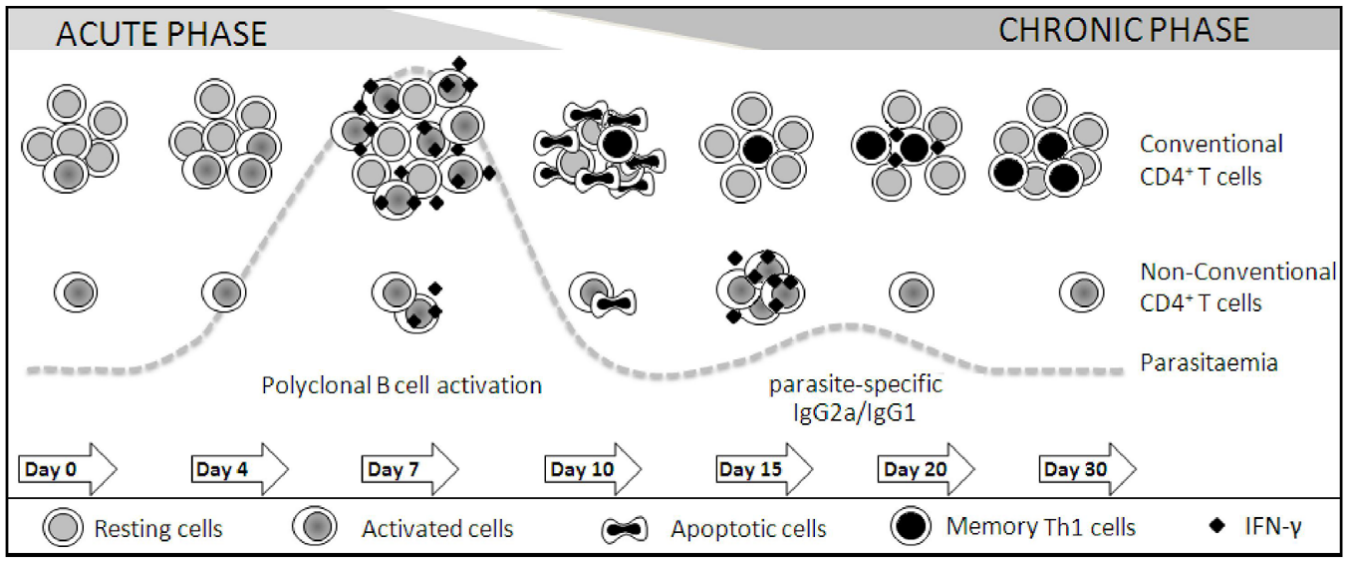
A model for the spleen CD4^+^ T cell response to blood-stages of *P. chabaudi* malaria. The spleen CD4+ T cell response to *P. chabaudi* infection develops in two consecutive phases concomitant with the acute and chronic parasitaemias. The early phase of the response begins with intense proliferation of a large proportion of conventional CD4+ T cells, which depends on the interaction with DC and class II MHC molecules and results in duplication of this population after a week of infection. The non-conventional CD4+ T cell population also duplicates during this period, but its low proliferative rate indicates that the majority of these cells undergo only a few rounds of cell division, or alternatively, they migrate from other tissues. This phase of the response culminates with production of high amounts of proinflammatory cytokines (and also with help to polyclonal Ig secretion), which is mostly dependent of conventional CD4+ T cells. The abrupt elimination of a great proportion of CD4+ T cells, together with a period of CD4+ T cell unresponsiveness, gives the opportunity to the development of acquired immunity during the late phase of the response. At this time, a large pool of CD4+ T cells is generated with the ability to promptly proliferate and produce IFN-γ when stimulated with iRBC (and also to cooperate with B cells for production of parasite-specific antibodies). Unexpectedly, the main contribution of non-conventional CD4^+^ T cells occurs in the beginning of the second phase of the response. Thus, according to this model, conventional CD4+ T cells are the main protagonists of this response from the onset of the disease, acting in parallel with non-conventional CD4+ T cells as a bridge between the innate and acquired immunity.

The results presented here indicate that the splenic CD4^+^ T cell response to blood-stages of *P. chabaudi* malaria develops in two consecutive phases as described in the model summarised in Figure 8. The early phase of the response may establish a bridge between innate and acquired immunity, as it rapidly provides large amounts of proinflammatory cytokines and helps B cells to secrete polyclonal Ig. The late phase of the response generates a large pool of parasite-specific CD4^+^ T cells that have the capacity to secrete IFN-γ and may cooperate with B cells for production of parasite-specific antibodies. The picture that emerges from our study is that conventional CD4^+^ T cells are the main protagonists of this response from the onset of the disease, whereas non-conventional CD4^+^ T cells play a secondary role. This is an interesting finding because it is generally accepted that non-conventional CD4^+^ T cells, such as NKT cells, are particularly shaped for early immune responses to infections while conventional CD4^+^ T cells are responsible for acquired immunity.

The central role of conventional CD4^+^ T cells in the early and late control of *P. chabaudi* malaria is evidenced by the fact that mice lacking class II MHC molecules develop significantly higher first parasitaemia peaks and prominent recrudescent peaks compared to WT mice, with 50% of them dying during the first month of infection. Similar results have been reported for *P. chabaudi* and *Plasmodium yoelii* malaria in two different models of class II MHC deficiency [36]. The 2-fold increase in the conventional splenic CD4^+^ T cell population a week after infection results from the vigorous proliferation of these cells, which requires the interaction with DC and class II MHC molecules. Confirming the secondary role of non-conventional CD4^+^ T cells in immunity to *P. chabaudi* infection, the contribution of CD1d-restricted CD4^+^ T cells is modest and limited to the second parasitaemia peak. Likewise, in *P. yoelii* malaria, the absence of CD1d-restricted CD4^+^ T cells leads to increased parasitaemias in the 4^th^ week of infection [37] but not earlier, while the acquisition of protective immunity is not affected [38]. The present study clarifies this issue by showing that the kinetic of CD1d-restricted CD4^+^ T cells correlates with their role in parasite control. Thus, the non-conventional CD4^+^ T cell population also duplicates during a week of infection but it represents only 10% of spleen CD4^+^ T cells of which 40% are iNKT cells. Different from the conventional CD4^+^ T cell population that achieves its maximum size on day 7 p.i., the peak of iNKT cell numbers per spleen occurred on day 15 p.i. when they represent 15% of CD4^+^ cells. As this population shows low levels of spontaneous and iRBC-stimulated proliferation, we postulate that the majority of non-conventional CD4^+^ T cells only undergo a few rounds of cell division or they migrate from other tissues.

The main consequence of the early phase of the spleen CD4^+^ T cell response to *P. chabaudi* malaria is the rapid induction of effector cells that are responsible for the secretion of intense but transient peaks of TNF-α and IFN-γ. These proinflammatory cytokines not only improve the ability of macrophages and DC to phagocytise and to present parasite antigens to T cells [19], [20], [39], [40], but also prime the immune system and thus favour host hyperresponsiveness to toll-like receptor (TLR) agonists [14], [40], [41], [42]. Conventional CD4^+^ T cells are required for the early production of TNF-α, while CD1d-restricted CD4^+^ T cells are dispensable for this response. In addition, conventional CD4^+^ T cells are the major source of IFN-γ a week after infection, starting its production at the same time as non-conventional CD4^+^ T cells. This observation has a parallel in the pre-erythrocytic stage of *P. yoelii* infection, where NK, NKT, γδ T and CD4^+^ T cells from the spleen simultaneously secrete IFN-γ but the latter cell population is the major source of production [43]. Moreover, similar to our analysis, in mice infected with *P. yoelii* sporozoites, IFN-γ is produced on day 5 p.i. but not earlier [43], raising the possibility that the ligands required for activation of non-conventional CD4^+^ T cells are not available during the first days of the disease.

The full activation of MZ and FO B cells at the early *P. chabaudi* infection depends on conventional CD4^+^ T cells, although some increase in CD69 expression occurs in B cells from mice lacking class II MHC molecules. Polyclonal Ig secretion is accompanied by downregulation of CD1d and CD21 in MZ B cells and CD23 in FO B cells. Low levels of CD1d in MZ B cells may result from disruption of the CD1d recycling machinery, as previously described for human DCs exposed to high titres of herpes simplex virus [44]. CD21 is expressed at low levels in various chronic infectious diseases, such as HIV (human immunodeficiency virus) infection, being attributed to continuous exposure of B cells to complement-coupled particles [45]. CD23 binds both CD21 and IgE, and through these interactions, regulates the synthesis of this antibody isotype [46], which has been associated with both protection and aggravation of malaria severity [47], [48]. It is surprising, however, that the entire population of MZ and FO B cells appears to be engaged in the early response to infection under the coordination of conventional CD4^+^ T cells. Although these cells are also required for induction of rapid secretion of polyclonal IgM, IgG2a and IgG1, low amounts of polyclonal IgM are produced during acute infection in mice lacking class II MHC molecules, indicating that part of this response is T-cell independent or dependent on non-conventional CD4^+^ T cells. However, the polyclonal Ig response is not affected by the absence of CD1d molecules. The IFN-γ-induced switch of polyclonal IgM to IgG2a [33], which according to our results is also dependent of conventional CD4^+^ T cells, may contribute to protection since antibody from this IgG isotype is the most effective to control blood-stage parasite growth [31].

With the control of acute parasitaemia, CD4^+^ T cell numbers per spleen rapidly decline, and in a few days, reach values that are lower than those in non-infected controls. This phenomenon is thought to be primarily due to CD4^+^ T cell apoptosis [49], [50], but other regulatory mechanisms may also ensure the end of the early phase of the response since the remaining cells become refractory to stimulation with iRBC or anti-CD3 mAb [51]. The end of the early response restricts the undesirable effects of overproduction of proinflammatory cytokines, such as the sepsis-like symptoms of acute malaria, and allows the development of a large population of parasite-specific CD4^+^ T cells, a process that occurs in parallel with a second wave of proliferation. This population increases with time up to a month of infection, but it progressively declines and reaches control levels when parasites are completed eliminated [52]. Despite the persistence of low levels of parasites during the late phase of the response, IFN-γ secretion is only detected at the second parasitaemia peak, although CD4^+^ T cells are able to produce high amounts of IFN-γ when stimulated *in vitro* with iRBC. Unexpectedly, iNKT cells are a major source of IFN-γ production at the beginning of the late phase of the response, when CD1d-restricted CD4^+^ T cells seem to be required for helping B cells to secrete low-affinity parasite-specific IgM and IgG antibodies. This may explain the incomplete control of parasitaemia recrudescence in mice lacking CD1d. Our results suggesting that only the low-affinity antibody response is impaired in the absence of CD1d-mediated interactions clarify contradictory studies on the role of CD1d-restricted CD4^+^ T cells for production of antibodies against glycosylphosphatidylinositol (GPI)-anchored parasite molecules [53], the secretion of parasite-specific high-affinity antibodies and the acquisition of full protective immunity strictly depending on conventional CD4^+^ T cells.

The present work adds basic information to the malaria literature and opens the perspective of future studies to unravel the molecular mechanisms involved in the early phase of the spleen CD4^+^ T cell response to *Plasmodium* infection. The engagement of several molecular pathways displaying either stimulatory or regulatory activities may be required for generating this response. The possibility that conventional CD4^+^ T cells recognise a diverse cohort of parasite components and self-antigens at different phases of the response is another interesting topic to be investigated. It is also of outstanding relevance to consider the influence of pre-erythrocytic stages on the spleen CD4^+^ T cell response to infection [43], a limitation of our study that only addresses the immune response to blood-stage parasites. We believe that clarifying these issues will help to develop new strategies to ameliorate the outcome of malaria by minimising the undesirable effects of the early CD4^+^ T cell response to *Plasmodium* infection.

## Supporting Information

Figure S1 **IFN-**γ **production by splenic CD8^+^ cells in** ***P. chabaudi*** **-infected WT, CD1d^-/-^ and I-A^b-/-^ mice.** (A) Dot plots showing intracellular IFN-γ in gated CD8+ cells on days 0, 4 and 7 of infection. Non-stimulated and anti-CD3-stimulated cell cultures are shown. Numbers in dot plots represent the means ± SD (n = 4–6) of IFN-γ+ cell percentages. (B) Numbers of CD8+IFN-γ+ cells per spleen on days 0, 4 and 7 of infection. Data represents the means ± SD (n = 4–6). In A-B, *, *p<*0.05, infected mice compared with non-infected mice; *ε*, *p<*0.05, CD1d-/- or I-Ab-/- mice compared with WT mice. Data are representative of three experiments.
(TIF)
