# Supplementary figures and images for: The Spleen CD4+ T Cell Response to Blood-Stage Plasmodium chabaudi Malaria Develops in Two Phases Characterized by Different Properties
Source: PLoS One. 2011 Jul 21;6(7):e22434. doi: 10.1371/journal.pone.0022434 (PMC3141041; doi:10.1371/journal.pone.0022434)

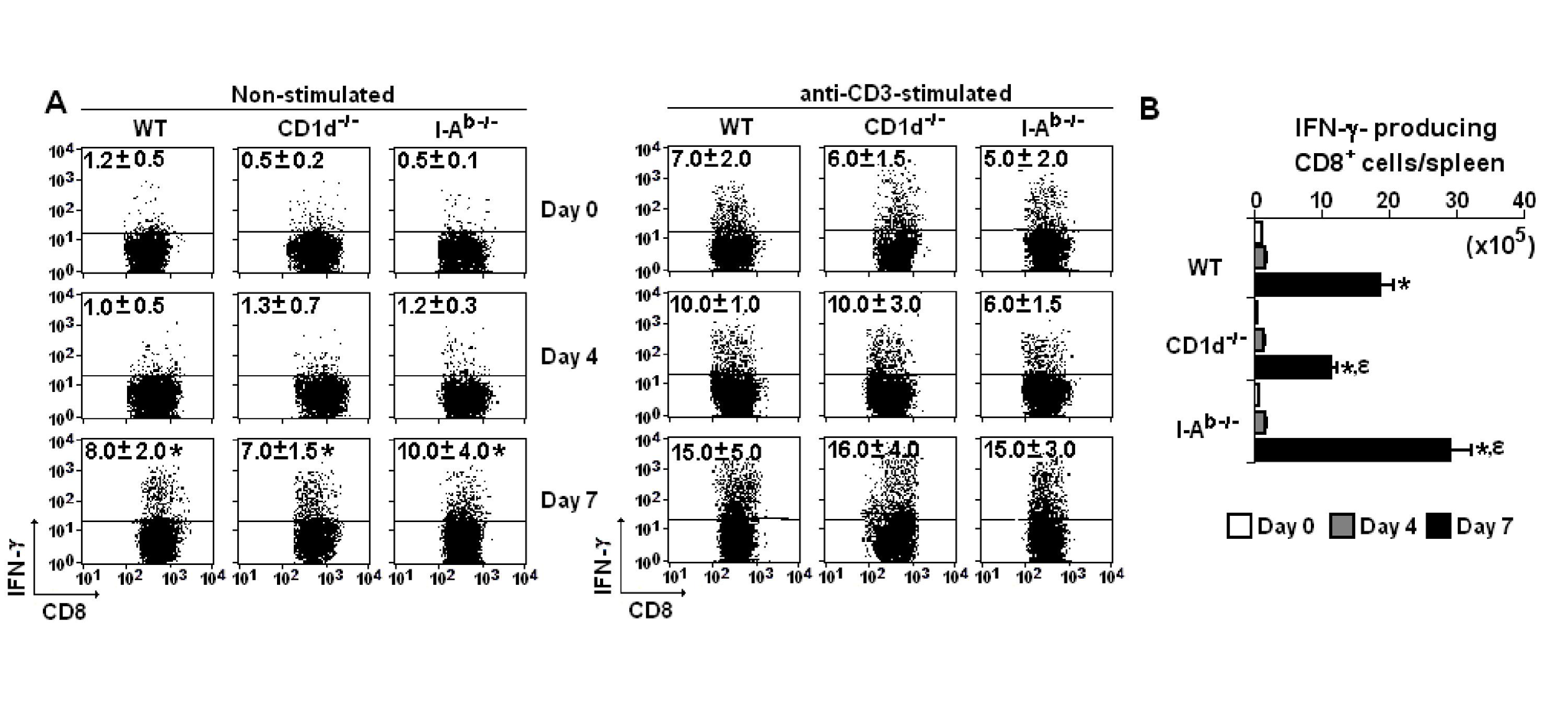

Supplement: Figure S1 — IFN-γ production by splenic CD8+ cells in P. chabaudi -infected WT, CD1d-/- and I-Ab-/- mice. (A) Dot plots showing intracellular IFN-γ in gated CD8+ cells on days 0, 4 and 7 of infection. Non-stimulated and anti-CD3-stimulated cell cultures are shown. Numbers in dot plots represent the means ± SD (n = 4–6) of IFN-γ+ cell percentages. (B) Numbers of CD8+IFN-γ+ cells per spleen on days 0, 4 and 7 of infection. Data represents the means ± SD (n = 4–6). In A-B, *, p<0.05, infected mice compared with non-infected mice; ε, p<0.05, CD1d-/- or I-Ab-/- mice compared with WT mice. Data are representative of three experiments. (TIF) [file pone.0022434.s001.tif]
